# *Sulcia* symbiont of the leafhopper *Macrosteles laevis* (Ribaut, 1927) (Insecta, Hemiptera, Cicadellidae: Deltocephalinae) harbors *Arsenophonus* bacteria

**DOI:** 10.1007/s00709-015-0854-x

**Published:** 2015-07-19

**Authors:** Michał Kobiałka, Anna Michalik, Marcin Walczak, Łukasz Junkiert, Teresa Szklarzewicz

**Affiliations:** Department of Developmental Biology and Morphology of Invertebrates, Institute of Zoology, Jagiellonian University, Gronostajowa 9, 30-387 Kraków, Poland; Department of Zoology, Faculty of Biology and Environmental Protection, University of Silesia, Bankowa 9, 40-007 Katowice, Poland

**Keywords:** Symbiotic microorganisms, *Sulcia*, *Nasuia*, *Arsenophonus*, Bacteriocytes, Deltocephalinae

## Abstract

The leafhopper *Macrosteles laevis*, like other plant sap-feeding hemipterans, lives in obligate symbiotic association with microorganisms. The symbionts are harbored in the cytoplasm of large cells termed bacteriocytes, which are integrated into huge organs termed bacteriomes. Morphological and molecular investigations have revealed that in the bacteriomes of *M. laevis*, two types of bacteriocytes are present which are as follows: bacteriocytes with bacterium *Sulcia* and bacteriocytes with *Nasuia* symbiont. We observed that in bacteriocytes with *Sulcia*, some cells of this bacterium contain numerous cells of the bacterium *Arsenophonus*. All types of symbionts are transmitted transovarially between generations. In the mature female, the bacteria *Nasuia*, bacteria *Sulcia*, and *Sulcia* with *Arsenophonus* inside are released from the bacteriocytes and start to assemble around the terminal oocytes. Next, the bacteria enter the cytoplasm of follicular cells surrounding the posterior pole of the oocyte. After passing through the follicular cells, the symbionts enter the space between the oocyte and follicular epithelium, forming a characteristic “symbiont ball.”

## Introduction

*Macrosteles laevis* is a representative of the subfamily Deltocephalinae, the largest group within the family Cicadellidae (Zahniser and Dietrich 2010, 2013). The Deltocephalinae leafhoppers are distributed worldwide. Most of them feed on xylem sap, using a needle-like mouthpart. Due to their feeding habitat, many of these insects are vectors of economically important plant diseases (caused by phytopathogenic viruses, bacteria, or fungi) (Brčak 1979).

The Deltocephalinae leafhoppers, like other plant sap-feeding hemipterans, harbor obligate symbiotic microorganisms, which synthesize and provide them with the amino acids missing in their diet (Douglas 1998, 2003, 2006; Baumann 2005).

Buchner (1965), basing his work on histological studies, distinguished two groups of symbionts: primary symbionts (also termed P-symbionts) and accessory symbionts (also termed secondary or facultative or S-symbionts). The primary symbionts provide their host insect with essential nutrients; therefore, their presence in the host body is necessary for its survival (i.e., proper functioning and reproduction) (Douglas 1998, 2006; Baumann et al. 2006; Moran and Dale 2006). The primary symbiosis is a result of a single long ago infection of an ancestor of the insect group by a free-living microorganism (review: Baumann 2005, 2006; Baumann et al. 2006). In consequence, such microorganisms are present in all representatives of this insect group. The obligate symbionts may be bacteria or yeast. The primary symbionts of Hemiptera: Sternorrhyncha (aphids, scale insects, whiteflies, and psyllids), Hemiptera: Cicadomorpha, Hemiptera: Fulgoromorpha, Hemiptera: Coleorrhyncha, and in some heteropterans are harbored in giant cells of mesodermal origin termed bacteriocytes (if bacteria) or mycetocytes (if yeast) (Buchner 1965; Baumann 2005, 2006; Baumann et al. 2006; Kuechler et al. 2013). These microorganisms are transovarially (vertically) transmitted between generations (see Buchner 1965 for further details).

Secondary symbionts may be present only in some populations of the species (review: Baumann 2005). Their presence in the insect body is the result of a more recent infection of an ancestor. The secondary symbionts may occur in bacteriocytes or in other types of insect cells (e.g., fat body cells), or free in hemolymph. These symbionts are not only transmitted transovarially but also between specimens of the same population (horizontally). The function of the S-symbionts was the object of many recent studies (e.g., Montlor et al. 2002; Oliver et al. 2003; Scarborough et al. 2005) which have shown that they impart specific properties to insects, such as the ability to survive heat stress or attack of parasitic hymenopterans or pathogenic fungi. In comparison to other hemipterans, there is still little data on the symbiotic systems (i.e., types of symbionts, their ultrastructure, distribution in the body cavity of insects, and mode of transovarial transmission) of Cicadomorpha. Earlier histological observations (Buchner 1965; Müller 1962) and more recent ultrastructural and molecular analyses (Moran et al. 2003; Takiya et al. 2006; Bressan et al. 2009; Noda et al. 2012; Bennett and Moran 2013; Ishii et al. 2013; Koga et al. 2013; Michalik et al. 2014; Szklarzewicz et al. 2015) have shown that these hemipterans may be hosts to several types of obligate symbionts. Recent molecular analyses (Takiya et al. 2006; Wu et al. 2006; McCutcheon et al. 2009; McCutcheon and Moran 2010) have revealed that in contrast to Sternorrhyncha, in which only primary symbionts are engaged in the synthesis of amino acids, in Cicadomorpha, all symbionts (termed by Takiya et al. 2006 as “coprimary symbionts”) synthesize these substances. Molecular analyses have revealed that members of Cicadomorpha and Fulgoromorpha (both formerly treated as Auchenorrhyncha) usually harbor the obligate Bacteroidetes bacterium “*Candidatus* Sulcia muelleri” (hereafter *Sulcia*) and one other kind of the coprimary symbionts belonging to the phylum Proteobacteria, e.g., “*Candidatus* Baumannia cicadellinicola” (hereafter *Baumannia*), “*Candidatus* Zinderia insecticola” (hereafter *Zinderia*), “*Candidatus* Nasuia deltocephalinicola” (hereafter *Nasuia*), or “*Candidatus* Hodgkinia cicadicola” (hereafter *Hodgkinia*) (Takiya et al. 2006; Urban and Cryan 2012; Bennett and Moran, 2013; Ishii et al. 2013; Koga et al. 2013). According to Moran et al. (2005) and Koga et al. (2013), the common ancestor of the Cicadomorpha and Fulgoromorpha was infected by the bacterium *Sulcia* and betaproteobacterial symbiont over 260 Ma ago. During the further evolution of some lineages of these hemipterans, the betaproteobacterium has been replaced by other bacteria, e.g., gammaproteobacterium *Baumannia* in some Cicadellinae and alphaproteobacterium *Hodgkinia* in cicadas. In some planthopper families and Deltocephalinae leafhoppers, the bacterial symbionts have been replaced by yeast symbionts (Noda 1977; Marzorati et al. 2006; Sacchi et al. 2008; Michalik et al. 2009).

To date, little is known about the symbiotic systems of the Deltocephalinae leafhoppers; however, the results of a few previous studies (Buchner 1925, 1965; Müller 1962; Marzorati et al. 2006; Sacchi et al. 2008; Wangkeeree et al. 2011; Noda et al. 2012; Bennett and Moran 2013; Ishii et al. 2013) suggest that these hemipterans are characterized by a large diversity of their symbionts. The aim of this study was to characterize the symbiotic system of the widely distributed species, *Macrosteles laevis*, in the Holarctic region*.*

## Material and methods

### Insects

**Fig. 1:**
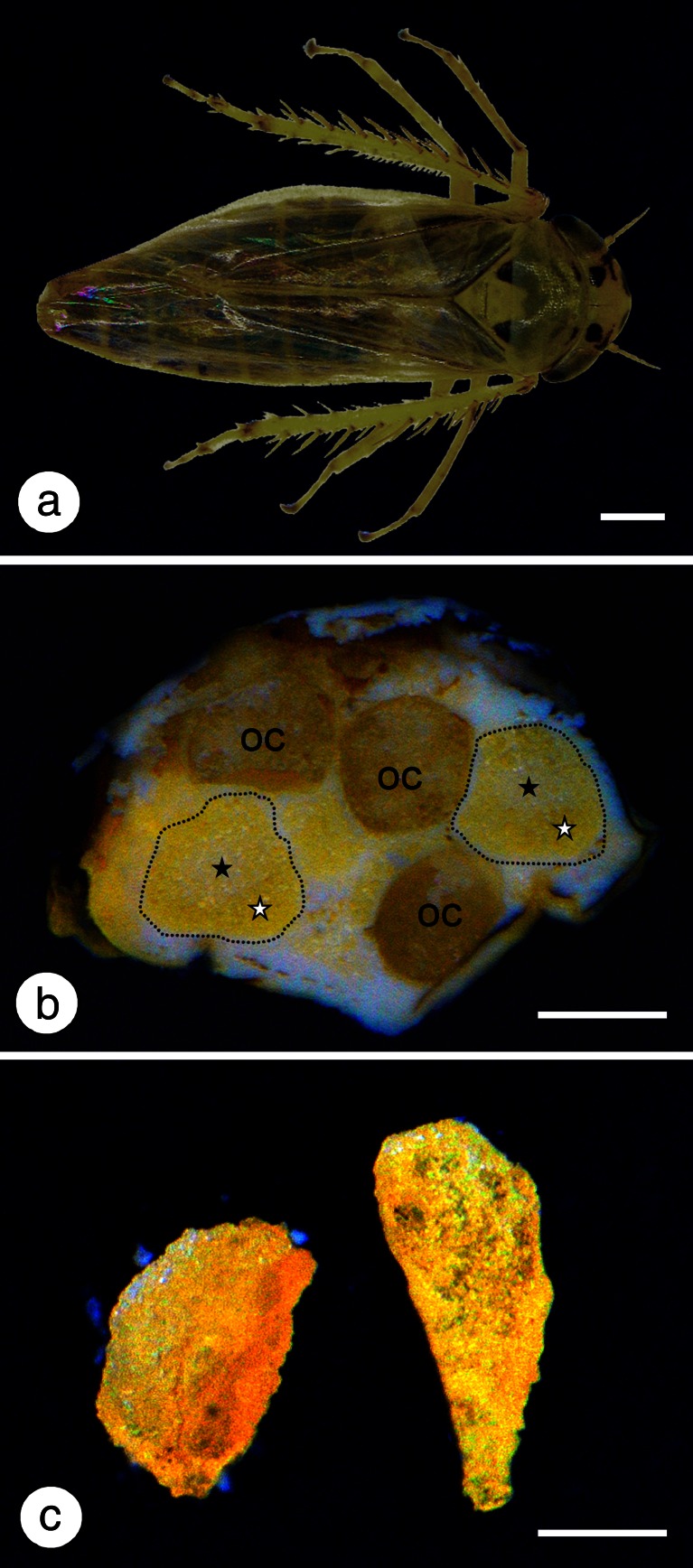
**a** Adult female of *Macrosteles laevis*. **b** Cross section through the abdomen of the adult female. Bacteriomes (*encircled with black dotted line*) are composed of two distinct types of bacteriocytes: peripherally located bacteriocytes with bacterium *Sulcia* (*white asterisk*) and centrally located bacteriocytes with bacterium *Nasuia* (*black asterisk*). Oocyte (*oc*). **c** Dissected bacteriomes. Stereomicroscope, *scale bar* = 0.5 mm

Adult males and females of *M. laevis* (Ribaut, 1927) (Fig. 1a) destined for histological and ultrastructural studies were collected from herbaceous plants in Kraków, Gliwice, Częstochowa, and Bielsko-Biała (in the south of Poland) from June to September during the years 2012–2014. Individuals destined for molecular analyses were collected in Częstochowa.

### DNA isolation

The bacteriomes were dissected from about 80 adult females of *M. laevis* and fixed in 100 % ethanol. DNA was extracted using Sherlock AX extraction kit (A&A Biotechnology) according to the manufacturer’s protocol with the following modification: before DNA extraction, bacteriomes were shaken for 60 s using a Beadbeater device with the addition of 0.3 ml distillated water, 0.3 ml lysis buffer L1.4, 20 μl proteinase K, and 0.5 g zirconia beads. DNA isolated from bacteriocytes was stored at −20 °C for further analyses.

### PCR, cloning, and sequencing

Bacterial 16S ribosomal DNA (rDNA) was amplified using the following primers: Late11B-all For: AGAAGGAGATATAACTAT GAG TTT GAT CCT GGC TCA G and Late11B-all Rev: GGAGATGGGAAGTCATTACG GCT ACC TTA CGA CTT. Next, PCR products were cloned into pLATE 11 plasmid vector using Thermo Scientific aLICator Ligation Independent Cloning and Expression System kit. The ligated mixture was transformed into competent cells *Escherichia coli* TOP10F which were prepared using *E. coli* Transformer kit (A&A Biotechnology). After 16 h, the occurrence of bacterial 16S rDNA was confirmed by a diagnostic PCR reaction with the following primers: pJET For: GCCTGAACACCATATCCATCC and pJET Rev: GCAGCTGAGAATATTGTAGGAGAT.

After restrictive analysis, the plasmids were isolated using Plasmid Mini Ax kit (A&A Biotechnology) and then six clones were sequenced. The determined nucleotide sequences were deposited in the GenBank database under the accession numbers KR337979–KR337981.

### Phylogenetic analysis

The phylogenetic analyses of symbiotic microorganisms were performed using the obtained sequences of 16S rDNA and homologous sequences downloaded from the GenBank database. The sequences were edited using BioEdit Sequence Alignment Editor 5.0.9 (Hall 1999); next, the sequence alignments were generated using ClustalX 1.8 (Thompson et al. 1997). All of the alignments were verified and then visually corrected. For the analyses, the appropriate nucleotide substitution model was first determined using the Modeltest 3.06 (Posada and Crandall 1998). The variable positions of the obtained 16S rDNA sequences were calculated using DnaSP software, whereas the base compositions of all analyzed genes were estimated using MEGA 6.0. software (Tamura et al. 2013). Phylogenetic analyses were conducted using Beast v 1.6.1 (Drummond and Rambaut 2007). The time of lineage divergence was estimated using the uncorrelated lognormal relaxed clock model. For each matrix, Beast was run using a Yule speciation process and two independent MCMC chains for 10,000,000 generations, sampling every 100th generation. The convergence to stationary and the effective sample size of the model parameters were checked using Tracer. The maximum clade credibility trees were built with TreeAnnotator (Drummond and Rambaut 2007). FigTree 1.4.0 software (Rambaut 2008) was used to visualize the results of phylogenetic analyses.

### Stereomicroscope observations

The specimens were fixed in 70 % ethanol. The adult specimens, sectioned abdomens, and dissected bacteriomes were photographed using a Nikon SMZ 1500 stereomicroscope.

### Light and electron microscopy analyses

For histological and ultrastructural studies, entire abdomens and dissected ovaries from about 80 individuals were examined. The specimens were fixed in 2.5 % glutaraldehyde in 0.1 M phosphate buffer (pH 7.4), rinsed in the same buffer with the addition of sucrose (5.8 g/100 ml), and postfixed in 1 % osmium tetroxide in the same buffer. Next, the material was dehydrated in a graded series of alcohols and acetone and embedded in epoxy resin Epon 812 (Serva, Heidelberg, Germany). Semithin sections (1 μm thick) were stained with 1 % methylene blue in 1 % borax and examined under light microscopes Leica DMR and Nikon Eclipse 80i. Ultrathin sections (90 nm thick) were contrasted with uranyl acetate and lead citrate and examined in Jeol JEM 2100 (Jeol, Japan) electron transmission microscope (TEM) at 80 kV.

## Results

### Molecular identification of symbiotic microorganisms of *M. laevis* and their phylogenetic analysis

Restrictive analysis and sequencing of the bacterial 16S rDNA clearly indicated the occurrence of three types of clones in the bacteriomes of *M. laevis.* One of them (signed as type 1) was dominant, whereas the other two types (signed as types 2 and 3) were less numerous. The nucleotide sequences obtained were analyzed and compared with the GenBank data using Blast. The Blast searches clearly demonstrated the affiliation of clones of type 1 to “*Candidatus* Sulcia muellerii” (Bacteroidetes) (99 % identity with 16S rDNA sequence of *Sulcia* symbiont of *Macrosteles quadrilineatus* [CP006060]. Type 2 clones were identified as a “*Candidatus* Nasuia deltocephalinicola” (Betaproteobacteria), with a top Blast hit to 16S rDNA sequence of *Nasuia* symbiont isolated from *Macrosteles sexnotatus* [AB795339]. Type 3 clones displayed the highest similarity to *Arsenophonus* symbiont of an aphid *Aphis melosae* [KF824523] and a scale insect *Ericerus pela* [JN990929].

The variation within the 16S rDNA sequences used in phylogenetic analyses was about 12 % variable sites and 8.5 % parsimony informative sites for *Sulcia* symbionts and about 47.5 % variable sites and 39.5 % parsimony informative sites for the *Nasuia* ones. The mean base compositions of the *Sulcia* symbionts were as follows: 30.9 % A, 25.1 % T, 17.3 % C, and 26.6 % G, while in the *Nasuia* symbionts, there were 33.9 % A, 26.8 % T, 14.9 % C, and 24.3 % G.

**Fig. 2:**
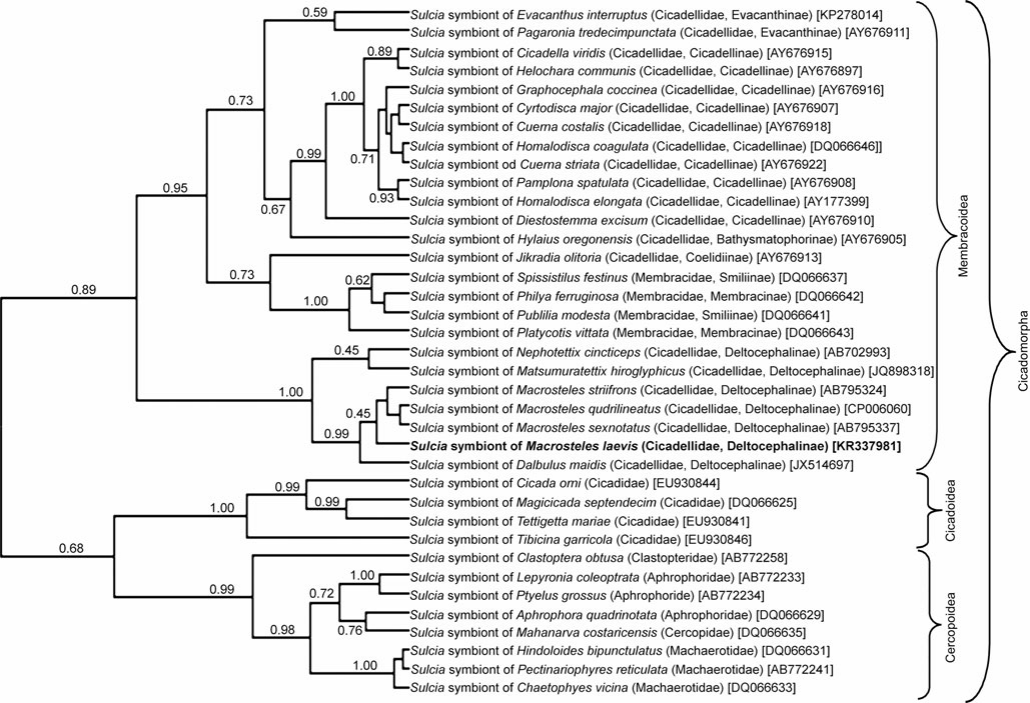
The phylogenetic tree based on 16S rRNA genes from *Sulcia* symbionts of extant Cicadomorpha was constructed using a Bayesian analysis. The *numbers* indicate posterior probabilities (*in brackets*—GenBank accession numbers of 16S rRNA genes)

**Fig. 3:**
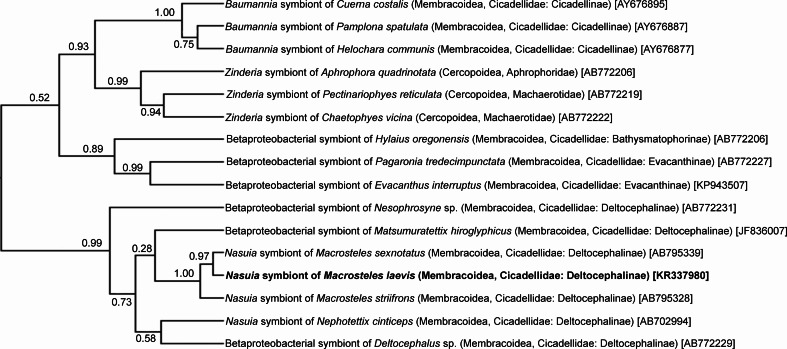
The phylogenetic tree based on 16S rRNA genes from proteobacterial symbionts of extant Cicadomorpha constructed using a Bayesian analysis. The *numbers* indicate posterior probabilities (*in brackets*—GenBank accession numbers of 16S rRNA genes)

The phylogenetic analysis of 16S rDNA sequences of *Sulcia* symbionts of various representatives of Cicadomorpha confirmed the affiliation of type 1 clones to Bacteroidetes symbionts. The phylogenetic tree (Fig. 2) indicates that the *Sulcia* symbionts of members of the Deltocephalinae subfamily create a monophyletic group within the symbionts of Membracoidea with 1.00 posterior probability values. The result of the phylogenetic analysis of 16S rDNA sequences from proteobacterial symbionts of the members of Cicadomorpha (Fig. 3) generally matches up with Blast search results. It was indicated that among proteobacterial symbionts of Cicadomorpha, *Nasuia* symbionts of representatives of Deltocephalinae subfamily are closely related and form a monophyletic group.

### Ultrastructure and distribution of symbiotic microorganisms

**Fig. 4:**
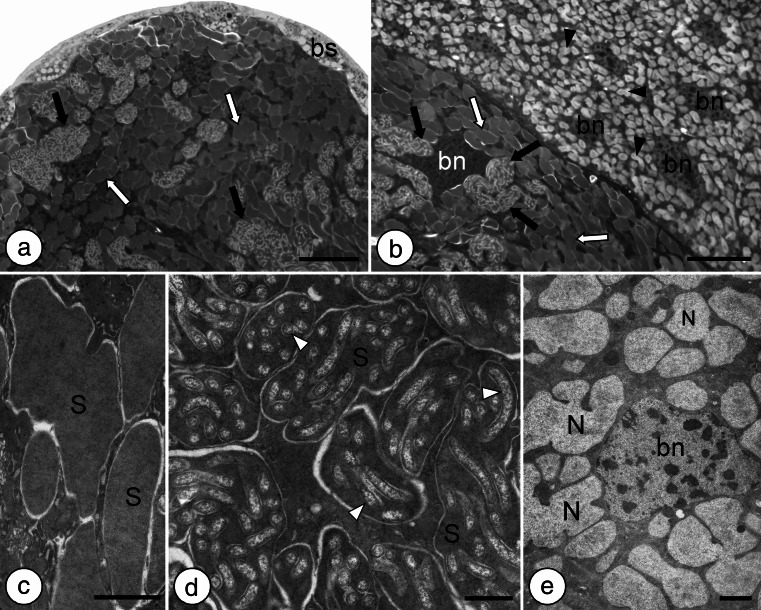
Organization of the bacteriome of *Macrosteles laevis*. **a** Fragment of the bacteriome comprising the bacteriocytes with bacterium *Sulcia* (*white arrows*) and bacterium *Sulcia* containing bacterium *Arsenophonus* (*black arrows*). Bacteriome sheath (*bs*). Methylene blue, *scale bar* = 20 μm. **b** Fragment of the bacteriome with bacteriocytes containing the bacterium *Sulcia* (*white arrows*), bacterium *Sulcia* with bacterium *Arsenophonus* inside (*black arrows*), and bacteriocytes with betaproteobacterial symbiont *Nasuia* (black arrowheads). Bacteriocyte nucleus (*bn*). Methylene blue, *scale bar* = 20 μm. **c** Fragment of the bacteriocyte filled with bacterium *Sulcia* (*S*). TEM, *scale bar* = 2 μm. **d** Fragment of the bacteriocyte filled with bacterium *Sulcia* (*S*) containing the bacterium *Arsenophonus* (*white arrowheads*). TEM, *scale bar* = 2 μm. **e** Fragment of the bacteriocyte filled with *Nasuia* (*N*). Bacteriocyte nucleus (*bn*). TEM, *scale bar* = 2 μm

Ultrastructural studies on symbionts of *M. laevis* revealed that within the body of each individual, three types of intracellular symbiotic bacteria occur. All the symbionts are harbored in the cytoplasm of bacteriocytes (Fig. 4a, b). Bacteriocytes form two large, yellow-colored structures termed bacteriomes that occur between the body wall and the gonads (Fig. 1b, c). The bacteriomes are surrounded by a thin sheath composed of a monolayered epithelium. These organs are present in both males and females; however, in females, they are much larger. Two types of bacteriocytes may be distinguished within each bacteriome: (1) the peripherally located bacteriocytes (Fig. 1b) filled with large, pleomorphic bacteria which stain intensely with methylene blue (Fig. 4a, b) and are electron-dense under an electron transmission microscope (Fig. 4c, d) and (2) the centrally located bacteriocytes (Fig. 1b) containing pleomorphic bacteria that stain with methylene blue less intensely (Fig. 4b) and are electron-translucent under an electron transmission microscope (Fig. 4e). Within the outer part of the bacteriome, in the cytoplasm of some large, pleomorphic bacteria, numerous long, rod-shaped bacteria occur (Fig. 4a, b, d).

The symbiotic bacteria present in the bacteriomes of *M. laevis* were identified based on the comparison of histological, ultrastructural, and molecular results. Based on the ultrastructural features of bacteria *Sulcia* and *Nasuia* (Bressan et al. 2009; Noda et al. 2012; Michalik et al. 2014; Szklarzewicz et al. 2015), the electron-dense pleomorphic bacteria localized in the peripheral part of the bacteriome have been identified as the Bacteroidetes species *Sulcia*, whereas electron-translucent pleomorphic bacteria occupying the inner part of the bacteriome as the betaproteobacterial symbiont *Nasuia.* The third type of symbionts, i.e., long, rod-shaped bacterium occurring inside the cells of *Sulcia*, has been identified as the gammaproteobacterial species *Arsenophonus.*

### Transovarial transmission of symbiotic microorganisms

All types of symbionts in the species studied are transovarially transmitted from mother to the offspring. The description of the consecutive stages of transovarial transmission of symbiotic bacteria was possible because the specimens of *M. laevis* were collected and fixed from June to September.

**Fig. 5:**
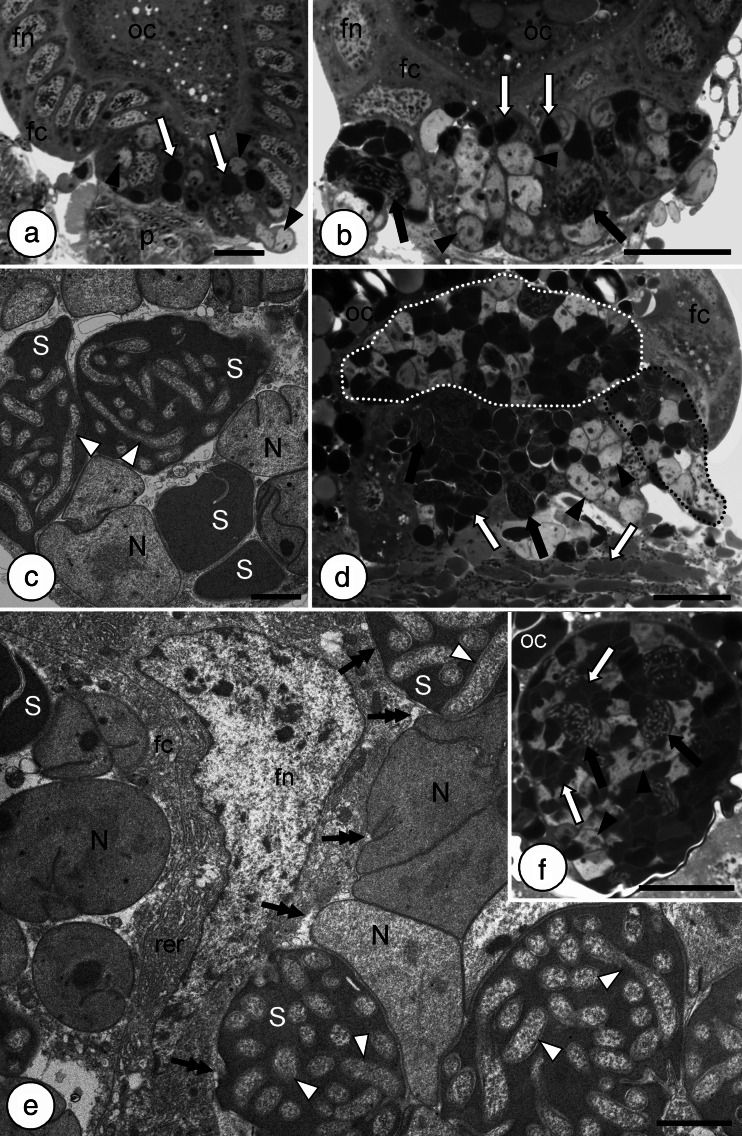
Consecutive stages of the infectioning of the terminal oocyte of the ovariole by symbiotic bacteria. **a**, **b** Posterior pole of the terminal oocyte (*oc*) (**a** early vitellogenic oocyte; **b** late vitellogenic oocyte) surrounded by a single layer of follicular cells (*fc*) (longitudinal section). Symbiotic bacteria enter the cytoplasm of follicular cells. *Sulcia* (*white arrows*), *Sulcia* with bacterium *Arsenophonus* inside (*black arrows*), *Nasuia* (*black arrowheads*), nucleus of follicular cell (*fn*), ovariolar stalk (pedicel) connecting the ovariole to the lateral oviduct (*p*). Methylene blue, *scale bar* = 20 μm. **c** High-magnification view of the follicular cell during the transmission of symbiotic bacteria. *Sulcia* (*S*), *Arsenophonus* (*white arrowheads*), *Nasuia* (*N*). TEM, *scale bar* = 2 μm. **d** Fragment of the terminal oocyte (*oc*) and enlarged follicular cells (*encircled with black dotted line*) filled with symbiotic bacteria. Note the lens-shaped accumulation of the bacteria in the perivitelline space (*encircled with white dotted line*) (longitudinal section). Follicular cells (*fc*), *Sulcia* (*white arrows*), *Sulcia* with bacterium *Arsenophonus* inside (*black arrows*), *Nasuia* (*black arrowheads*). Methylene blue, *scale bar* = 20 μm. **e** High-magnification view of follicular cell (*fc*) filled with symbiotic bacteria and perivitelline space (*black double arrows*) with the accumulation of symbionts. *Sulcia* (*S*), *Arsenophonus* (*white arrowheads*), *Nasuia* (*N*), nucleus of follicular cell (*fn*), cisternae of rough endoplasmic reticulum (*rer*). TEM, *scale bar* = 2 μm. **f** The accumulation of symbionts (“symbiont ball”) in the perivitelline space in the deep invagination of oolemma. Oocyte (*oc*), *Sulcia* (*white arrows*), *Sulcia* with bacterium *Arsenophonus* inside (*black arrows*), *Nasuia* (*black arrowheads*). Methylene blue, *scale bar* = 20 μm

**Fig. 6:**
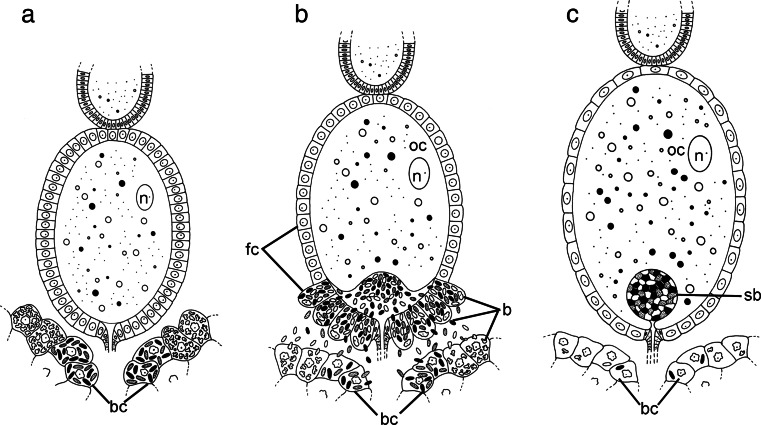
Schematic representation of the transovarial transmission of symbionts in *Macrosteles laevis*. **a** The terminal oocytes are accompanied by bacteriocytes. **b** Symbiotic bacteria leave the bacteriocytes, invade follicular cells surrounding posterior pole of the terminal oocyte, and enter the space between the oocyte and follicular epithelium. **c** Symbiotic bacteria accumulate in the deep invagination of the oolemma forming a characteristic “symbiont ball.” Symbiotic bacteria (*s*), bacteriocyte (*bc*), follicular cells (*fc*), oocyte (*oc*), oocyte nucleus (*n*), “symbiont ball” (*sb*)

The ovaries of *M. laevis* are composed of seven ovarioles of telotrophic type (for further details concerning the classification and organization of insect ovarioles, see Büning 1994; Biliński 1998) that via ovariolar stalks (pedicels) join with lateral oviduct. Each ovariole contains several linearly arranged oocytes that are surrounded by a single layer of follicular cells (Figs. 5a and 6a). In the females containing ovaries with terminal oocytes in the stage of early vitellogenesis, bacteria *Nasuia*, bacteria *Sulcia*, and bacteria *Sulcia* filled with bacteria *Arsenophonus* leave the cytoplasm of bacteriocytes and begin to invade ovarioles. Before migrating from the bacteriocytes into the gonads, the bacteria change their shape from irregular into roughly spherical (Fig. 5a, e). The symbiotic microorganisms enter the follicular cells in the region of the posterior pole of terminal oocytes (Figs. 5a and 6b) and temporarily accumulate in their cytoplasm (Figs. 5b–e and 6b). During this invasion of symbionts, the volume of infected follicular cells significantly increases (Figs. 5b, d and 6b). Next, the bacteria leave the follicular cells and enter the perivitelline space (i.e., space between the oocyte and follicular epithelium) (Figs. 5d and 6b). The symbionts assemble in the invagination of the oolemma of the posterior pole of the oocyte initially in the lens-like form (Figs. 5d and 6b) and finally in the spherical form termed “symbiont ball” (Figs. 5f and 6c). The bacteria residing in the “symbiont ball” change their shape from spherical to polygonal and closely adhere to one another (Figs. 5d–f and 6c). When the infection of the ovariole is near termination, the number of organelles in the cytoplasm of follicular cells of the posterior pole of the oocyte, especially the cisternae of rough endoplasmic reticulum and Golgi complexes, increases greatly (Fig. 5e). These changes in the ultrastructure of follicular cells are associated with the production of egg envelopes.

## Discussion

Müller (1962) and Buchner (1965), on the basis of histological observations of several species of the Deltocephalinae, distinguished two types of their symbionts termed *a*-symbionts and *t*-symbionts. Results of recent molecular studies on symbionts living in several species belonging to three genera, *Macrosteles*, *Nephotettix*, and *Matsumuratettix*, have shown that they harbor the bacterium *Sulcia* and the betaproteobacterial symbiont *Nasuia* which correspond to *a*-symbionts and *t*-symbionts (respectively) (Wangkeeree et al. 2011; Noda et al. 2012; Bennett and Moran 2013; Ishii et al. 2013; this study). Thus, the above facts indicate that *Macrosteles*, *Nephotettix*, and *Matsumuratettix* retained the ancestral dual symbiotic system (see “Introduction”). Molecular phylogenetic analyses (see Figs. 2 and 3) have revealed that both *Sulcia* and betaproteobacterial symbiont *Nasuia* living in *M. laevis* form well-defined clades with *Sulcia* and betaproteobacterial symbionts of other Deltocephalinae leafhoppers.

In contrast to the dual “*Sulcia-*betaproteobacteria” system, the bacteriocyte-associated bacterium *Cardinium* (Bacteroidetes) and mycetocyte-associated yeast-like symbionts are present in another member of the Deltocephalinae, *Scaphoideus titanus* (Marzorati et al. 2006; Sacchi et al. 2008). Furthermore, in the body of some Deltocephalinae leafhoppers, additional bacteria, e.g., *Wolbachia*, *Rickettsia*, *Burkholderia*, and *Diplorickettsia*, have been identified (Noda et al. 2012; Ishii et al. 2013). Our ultrastructural and molecular analyses have indicated that in *M. laevis* besides *Sulcia* and *Nasuia*, inside *Sulcia* cells, numerous gammaproteobacteria *Arsenophonus* are present. Thus, the state of symbiosis in the representatives of the Deltocephalinae appeared even more complex.

The presence of *Arsenophonus* inside the cells of the bacterium *Sulcia* is of special interest because such “an internal symbiosis” is very rare phenomenon within insects. To date, “symbionts within symbionts” have been reported only for some members of the scale insect family Pseudococcidae (mealybugs) (von Dohlen et al. 2001; Thao et al. 2002; Kono et al. 2008; Gatehouse et al. 2011; McCutcheon and von Dohlen 2011) and for the green leafhopper, *Cicadella viridis* (Cicadomoropha, Cicadellidae: Cicadellinae) (Michalik et al. 2014). Molecular analyses have revealed that in both mealybugs and in *C. viridis*, the internalized bacteria are closely related to the gammaproteobacterium *Sodalis glossinidius* that is the secondary symbiont of tsetse fly (Gatehouse et al. 2011; Michalik et al. 2014). In contrast to the closely related internal symbionts of mealybugs and *C. viridis*, their host bacteria are characterized by a different systematic affinity: in mealybugs, they belong to the class Betaproteobacteria of the phylum Proteobacteria (von Dohlen et al. 2001; Baumann et al. 2002; Gatehouse et al. 2011), and in *C. viridis*, to the phylum Bacteroidetes (Michalik et al. 2014). Additionally, in mealybugs, *Sodalis*-like bacteria are always harbored in the cytoplasm of betaproteobacterial symbionts, whereas in *C. viridis*, they may occur individually in the cytoplasm of their own bacteriocytes or may coreside in bacteriocytes with the bacterium *Sulcia*. It has been observed that during the coexistence of both bacteria in the cytoplasm of the same bacteriocyte, *Sodalis*-like bacteria enter the cytoplasm of *Sulcia*. In consequence, bacteria *Sulcia* containing internalized *Sodalis*-like bacteria are transmitted to the next generation. On the basis of the above observations, Michalik et al. (2014) suggested that the presence of *Sodalis*-like bacteria represents the youngest stage of association (in statu nascendi). Our ultrastructural observations have revealed that in *M. laevis*, as in mealybugs, the internalized bacteria never occur individually, but always (i.e., in bacteriocytes, during the infectioning of ovaries, and in the ovarioles) live inside the cells of the bacterium *Sulcia*. This fact suggests that just as in the case of mealybugs (Thao et al. 2002), the association of both symbionts and *M. laevis* is of more ancient origin than in *C. viridis*.

The gammaproteobacterium *Arsenophonus* is widespread within arthropods (Nováková et al. 2009). Results of numerous studies have shown that this bacterium may play various functions—it may be primary or secondary symbionts of blood- or plant sap-sucking insects (Thao et al. 2000; Russell et al. 2003; Trowbridge et al. 2006; Jousselin et al. 2013), plant pathogens vectored by planthoppers (Bressan 2014), or male killers in the parasitic wasp *Nasonia vitripennis* (Huger et al. 1985; Gherna et al. 1991). It has been also revealed that *Arsenophonus* may be horizontally and vertically transmitted to the next generation (Jousselin et al. 2013); however, the mode of transovarial transmission of this microorganism is poorly understood. To our knowledge, the occurrence of the bacterium *Arsenophonus* inside the cells of other bacterium has never been described in any insects. It is very difficult to interpret this phenomenon, but it may be assumed after von Dohlen et al. (2001) and Michalik et al. (2014) that, like the bacterium *Sodalis* in mealybugs and in the green leafhopper *C. viridis*, the bacterium *Arsenophonus* may be protected in the cytoplasm of other bacteria from the immune system of the host insect. This hypothesis is supported by observations that *Arsenophonus* may play various roles—from symbiotic to parasitic. Thus, its association with host insects may be more or less ancient. This, in turn, suggests that the more recently acquired bacterium *Arsenophonus* retained their virulence factors which might be detected by the insect immune system.

It should be stressed that *Arsenophonus* has not been found in the two species of *Macrosteles* collected in Japan: *Macrosteles striifrons* and *M. sexnotatus* (Ishii et al. 2013), but it has been detected in the species *M. quadrilineatus* collected in the USA (Bennett and Moran 2013). According to Bennett and Moran (2013), *Arsenophonus* does not represent an obligate symbiont of *M. quadrilineatus*. The distribution of *Arsenophonus* in the body of *M. quadrilineatus* as well as the mode of their transmission from mother to offspring remains unknown. The occurrence of *Arsenophonus* only in some species of *Macrosteles* and its absence in geographically isolated species strongly support the view of a recent association between these hemipterans and *Arsenophonus.* To verify this hypothesis, further molecular and ultrastructural studies on symbionts in other *Macrosteles* species are needed.

Earlier papers (Buchner 1925, 1965; Müller 1962) and more recent studies (Cheung and Purcell 1999; Sacchi et al. 2008; Michalik et al. 2014; this study) indicated that in Cicadomorpha, the symbiotic microorganisms migrate to the ovaries in a similar way, i.e., via the cytoplasm of follicular cells. Thus, in spite of the enormous diversity of types of symbionts living in Cicadomorpha, the mode of their transmission from mother to the offspring seems to be more uniform.
